# Biocontrol and plant growth promoting potential of phylogenetically new *Streptomyces* sp. MR14 of rhizospheric origin

**DOI:** 10.1186/s13568-019-0849-7

**Published:** 2019-08-09

**Authors:** Talwinder Kaur, Riveka Rani, Rajesh Kumari Manhas

**Affiliations:** Department of Microbiology, Guru Nanak Dev University, Amritsar, Punjab 143005 India

**Keywords:** Fungal phytopathogens, Biocontrol, Plant growth promotion, Rhizosphere, *Streptomyces*, Fusarium wilt

## Abstract

The detrimental effects of synthetic fungicides have increased the emphasis for biological control as an effective and safe sustainable alternative method. In the present work, a potent rhizospheric actinobacterium MR14 showed broad spectrum antifungal and plant growth promoting activities indicating the potential to fulfill the need. Phylogenetic analysis confirmed that the isolate could be assigned as new species of the *Streptomyces*, coded as *Streptomyces* sp. MR14. It formed clade with *Streptomyces daghestanicus* but with very low bootstrap value (14%). The MR14 supernatant showed potent antagonistic activity against 13 different tested fungal phytopathogens. The most and least sensitive fungal phytopathogens were found to be *Pyricularia oryzae* and *Fusarium oxysporum* with inhibition zones of 31 mm and 11 mm, respectively. The antifungal metabolites produced by strain MR14 were thermostable, photostable, and remained active at extreme acidic and neutral pH. In pot experiments, the *Streptomyces* sp. MR14 cells, supernatant and extract significantly suppressed Fusarium wilt caused by *Fusarium moniliforme* in tomato plants. Various growth parameters such as shoot and root lengths, and plant fresh and dry weights were significantly enhanced by 19.65 to 321.62% over the pathogen infested plants only. The treatment with culture cells/supernatant/extract in the rhizosphere soil also reduced the microbial count as compared to control. In addition, the strain also possessed plant growth promoting potential which was indicated by the increase in various agronomic traits from 3.64 to 116.88%. This study provided a scientific validation that the new rhizobacterium *Streptomyces* sp. MR14 could be further developed as bioformulation, exhibiting biocontrol and plant growth promoting capabilities.

## Introduction

Worldwide rapidly increasing population is one of the greatest challenges for the agriculture field. This global struggle becomes more disastrous with the loss of crop yield due to the phytopathogens (bacterial, fungal, pests, nematodes etc.) especially, various fungal phytopathogens (Oerke 2006). The current strategies employed to control plant fungal diseases are mainly the application of synthetic fungicides including triazoles and acylalanines, and the development of resistant varieties (Emmert and Handelsman 1999). However, the arbitrary use of synthetic fungicides has resulted in the development of fungicide resistance in the pathogens, environmental pollution, ecological imbalance in the soil and adverse effects on human health and beneficial microflora (Fox et al. 2007; Thind 2008; Ntalli and Menkissoglu-Spiroudi 2011). So, their use is being restricted in several countries which further necessitate the quest for biological control as an effective and long lasting alternative to control various soil-borne fungal diseases. The specificity to the host plant, adaptiveness of most biocontrol agents (BCAs) to the environment, the involvement of several mechanisms of disease suppression by a single microorganism and the complex organismal interactions contribute to the belief that the biological control is more effective and durable than chemical fungicides (Sharma and Sharma 2008; Manhas and Kaur 2016).

Among natural sources, microorganisms always have been accounted as the solution to every problem and now become the center of intensive research globally. Thus far, various microbial antagonists of rhizospheric origin including species of *Bacillus*, *Pseudomonas*, *Streptomyces*, *Trichoderma* and nonpathogenic *Fusarium* have been effectively used as BCAs (Heydaria and Pessarakli 2010; Faheem et al. 2015). They are also used for promotion of the plants by enhancing the nutrient availability to the plants (Gopalakrishnan et al. 2011).

Such novel microbial species having biocontrol and plant growth promoting capabilities mainly reside at interface between roots of higher plants and soil i.e. rhizosphere and benefit the host through a number of mechanisms (Heydaria and Pessarakli 2010; Shobha and Kumudini 2012). In well-studied rhizosphere of a healthy plant, availability of nutrients and organic materials derived from the root exudates become the driving force for the abundance of diverse microbial community (Raaijmakers et al. 2009). The complex plant–microbe interactions such as biogeochemical cycling of nutrients, protection from phytopathogens and production of various growth promoting hormones affect the plant health and function which in turn improve the global productivity (Berendsen et al. 2012; Philippot et al. 2013).

Among the rhizospheric microbial community, the immense importance is given to the genus *Streptomyces* for the production of vast array of compounds with pharmaceutical and agricultural importance (Prabavathy et al. 2006; Palaniyandi et al. 2013). Spore formation by these filamentous and Gram-positive rhizobacteria also offers an additional advantage to be developed as plant growth improving agents through formulations (Emmert and Handelsman 1999; Shobha and Kumudini 2012). Some species of *Streptomyces* have already been successfully developed into formulations to control fungal phytopathogens on various crops. Commercially available wettable formulation Mycostop consisting of spores and mycelium of *Streptomyces griseoviridis* has been extensively used in Europe and North America for the protection of ornamental and vegetable crops (Tahvonen and Avikainen 1987). Another non commercial wettable formulation prepared from *Streptomyces* sp. Di-944 prevents damping off disease of tomato caused by *Rhizoctonia solani* (Sabaratnam and Traquair 2002). However, more novel species of *Streptomyces* are still waiting to be identified and developed as biocontrol and plant growth promoting agents.

Keeping in view the importance of *Streptomyces* as biocontrol agents, a potent streptomycete isolate, designated as MR14, exhibiting antifungal and plant growth promoting activities was isolated from the rhizosphere soil of mustard (*Brassica nigra*), collected from Amritsar (India). The objective of this study was to characterize the isolate MR14 using polyphasic approach and to assess the in vivo effect of the isolate to control Fusarium wilt caused by *F. moniliforme* on the tomato plants. Further, the in vivo potential of strain on plant growth was evaluated by observing its effect on various agronomic traits in tomato plant.

## Materials and methods

### Sample collection and isolation of *Streptomyces* sp. MR14

The strain MR14 (MTCC 12924) was isolated from soil sample collected from rhizosphere of mustard (*B. nigra*) plant, from the designated site MR-ASR (31.63°N 74.87°E), situated in Amritsar, Punjab (India) (Kaur et al. 2013). The isolate exhibited antagonistic activity against different fungal phytopathogens and also various plant growth promoting activities such as production of indole acetic acid, siderophore and ammonia. The isolate MR14 was maintained on Starch Casein Nitrate Agar medium (SCNA; g/L): starch 10.0, casein 0.3, KNO_3_ 2.0, NaCl 2.0, K_2_HPO_4_ 2.0, MgSO_4_·7H_2_O 0.05, CaCO_3_ 0.02, FeSO_4_·7H_2_O 0.01 and agar 20.0) slants at 4 °C in the refrigerator and as spore suspensions in 20% (*v/v*) glycerol at − 70 °C in an ultra-low temperature freezer.

### Test microorganisms

Various phytopathogenic fungi used in this study viz. *Alternaria brassicicola* (MTCC 2102), *Colletotrichum acutatum* (MTCC 1037), *Cladosporium herbarum* (MTCC 351), *Fusarium oxysporum* (MTCC 284), *Alternaria solani* (MTCC 2101), *Pyricularia oryzae* (MTCC 1477) and *Fusarium oxysporum* f.sp. *dianthi* (MTCC 6659) were obtained from Microbial Type Culture Collection (MTCC) and Gene Bank, Institute of Microbial Technology (IMTECH), Chandigarh, India. *Cercospora beticola* (KJ461435), *Exserohilum* sp., *Fusarium moniliforme*, *Colletotrichum gloeosporioides*, *Alternaria mali* and *Alternaria alternata* were isolated in the laboratory. All the fungal cultures were maintained on Potato Dextrose Agar (PDA) slants at 4 °C.

### Characterization and identification of isolate MR14 using polyphasic approach

#### Morphological, biochemical and physiological characterization

The isolate MR14 was characterized on the basis of cultural characteristics (sporulation, color of the aerial and substrate mycelia and soluble pigment production in the medium) as per methods prescribed in International *Streptomyces* Project (Shirling and Gotllieb 1966). The isolate was inoculated on SCNA and different ISP media (ISP-1, ISP-2, ISP-3, ISP-4, ISP-5, ISP-6 and ISP-7) for 7 days at 28 °C. Morphological properties of the isolate were observed by the light microscope (Olympus) at 100X, and micro-morphological properties such as spore chain and spore surface morphologies were determined by scanning electron microscopy (Carl Zeiss model EVOLS 10). Assimilation of variety of sugars as carbon sources was studied according to Shirling and Gotllieb (1966). d-glucose, xylose, lactose, maltose, cellulose, glycerol, starch, sucrose, fructose and l-inositol (1%) (HiMedia, India) were added to the basal medium after filter sterilization. The capability of the isolate to produce industrially important enzymes (amylase, protease, lipase, cellulase, chitinase, pectinase, gelatinase, urease, catalase and oxidase), and H_2_S, and utilization of citrate were determined according to Cowan and Steel (1965). Indole production and Methyl Red and Voges Proskauer (MR––VP) tests were performed as recommended by Holding and Collee (1971). Physiological tests were performed by growing the strain on SCNA at different temperatures (20–50 °C) and different NaCl concentrations (0–20% *w/v*). Analysis of isomers of diaminopimelic acid (DAP) in the cell wall and sugars in the whole-cell hydrolysate was done according to the method given by Lechevalier and Lechevalier (1970).

#### Genomic characterization based on 16S rRNA gene sequencing

For 16S rRNA gene sequencing, the genomic DNA of the isolate was extracted following the method given by Marmur (1961), and then amplification of the 16S rRNA gene sequence was performed by polymerase chain reaction (PCR) using primers 27f (5′-AGAGTTTGATCCTGGCTCAG-3′) and 1492r (5′-AGAAAGGAGGTGATCCAGGC-3′). The obtained PCR product was purified using QIA quick gel extraction kit (Qiagen, Germany). The purified PCR product got sequenced from Institute of Microbial Technology (IMTECH), Chandigarh, India. Using the EzTaxon server (http://www.ezbiocloud.net/), identification of phylogenetic neighbors and calculation of pair wise 16S rRNA gene sequence similarities were achieved (Chun et al. 2007). The almost complete sequence (1434 bp) of isolate MR14 and the sequences of phylogenetic neighbors were aligned using Clustal W Program. Phylogenetic trees were constructed according to the neighbor joining and maximum-parsimony algorithms using bootstrap values based on 1000 replications with the MEGA 6.0 software (Felsenstein 1985; Tamura et al. 2013).

### In vitro antifungal activity profile of *Streptomyces* sp. MR14 against fungal phytopathogens

For the inoculum preparation, the growth of 7 days old streptomycete MR14 was transferred aseptically into the seed medium (SCN broth) and incubated for 48 h at 28 °C on an orbitek rotary shaker at 180 rpm. Fermentation was carried out by inoculating the production medium (SCN broth) with seed culture (2%) and incubated at 28 °C for 1 week under agitation at 180 rpm. After every 24 h, the flasks were harvested and the culture broth was centrifuged at 10,000 rpm for 20 min at 4 °C to separate the biomass. The biomass was dried at 60 °C for 2 days, weighed and expressed in mg on dry weight basis.

The remaining cell free culture supernatant was used to check the activity against different fungal phytopathogens by agar well diffusion method (Bauer et al. 1966). The PDA (Potato Dextrose Agar; Hi-Media) plates, inoculated with test fungi (100 μL; 10^5^  spores/mL), were punctured with sterile cork borer to make wells (9 mm), and 200 μL of culture supernatant was added to each well under aseptic conditions. Prior to incubation, the plates were kept at 4 °C for 30 min for diffusion of the antifungal compounds. Then the PDA plates were incubated at 28 °C for 4–5 days. The antifungal activity of the isolate was detected as clear zones of inhibition around wells, measured in millimeters.

### Stability of antifungal compounds in culture supernatant of *Streptomyces* sp. MR14

To check the thermostability of the antifungal metabolites produced by *Streptomyces* sp. MR14, culture supernatant was kept at different temperatures (− 20 °C, 37 °C, 50 °C, 70 °C, 100 °C, autoclaving) for one hour. Photostability was tested by exposing the supernatant separately to UV and sunlight for 1 h. To investigate the enzyme stability, culture supernatant was treated with 0.1 mg/mL proteinase K and trypsin at 37 °C for 60 min. The effect of pH on antifungal activity was investigated by adjusting the pH of culture supernatant at 2.0 and 14.0, followed by incubation for 1 h at 28 °C. All the treated samples were then checked for the residual activity using *F. moniliforme*. The shelf life of the antifungal metabolites at 4 °C was determined by storing the culture supernatant in refrigerator and checking the antifungal activity at regular intervals of time for 6 months.

### Extraction of bioactive metabolites

The extraction of antifungal metabolites was done using solvent–solvent extraction technique. The culture supernatant of *Streptomyces* sp. MR14 was extracted twice in the ratio of 1:1 (culture supernatant: solvent) with six different solvents of a wide range of polarity, namely, n-butanol, methanol, ethyl acetate, chloroform, diethyl ether and hexane. The organic phase was concentrated to dryness using rotavapor (BUCHI). The obtained crude extracts were redissolved in respective solvents and tested for their antifungal activity using agar disc diffusion assay (Bauer et al. 1966). As the maximum recovery of antifungal metabolites was obtained in ethyl acetate further work was done using ethyl acetate extract of MR14. For in vivo biocontrol assay, the ethyl acetate extract of MR14 (250 µg/mL) was dissolved in 0.5% DMSO.

### In vivo biocontrol of *F. moniliforme* causal organism of Fusarium wilt by *Streptomyces* sp. MR14 and its effect on plant growth promotion in tomato plants

In vivo pot experiment was conducted in the month of 10th Oct–25th Nov, 2017 at Guru Nanak Dev University, Amritsar using soil drenching method. The aim was to investigate the potentiality of the culture cells, culture supernatant as well as culture extract of *Streptomyces* sp. MR14 to control the *F. moniliforme* (causal fungal phytopathogen of Fusarium wilt) and promote various plant growth traits. Seeds of tomato (*Solanum lycopersicum* Mill., variety Pusa Ruby, susceptible to *F. moniliforme*) were sown in sterilized soil at 28 ± 2 °C for 1 month. The pots (8 cm diameter) containing 100 g of autoclaved soil were divided into 8 groups and each group was given different treatment. Group 1 (C) control: soil was treated with 10 mL water only, Group 2 (P) fungal pathogen: soil was infested with 10 mL of fungal *F. moniliforme* spore suspension (1 × 10^6^ spores/mL); Group 3 (CC + P) culture cells and pathogen: soil was inoculated with 10 mL of pathogen spore suspension and 10 mL of *Streptomyces* sp. MR14 culture cell suspension (1 × 10^6^ cells/mL) prepared in autoclaved water; Group 4 (CS + P) culture supernatant and pathogen: soil was inoculated with 10 mL of *F. moniliforme* suspension and 10 mL of culture supernatant obtained from 4 days old fermentation broth of *Streptomyces* sp. MR14; Group 5 (CE + P) culture extract and pathogen: soil was inoculated with 10 mL of fungal spore suspension and 10 mL of culture extract of *Streptomyces* sp. MR14 dissolved in water (250 µg/mL); Group 6 (CC) culture cells only: soil was treated with 10 mL of cell suspension (1 × 10^6^ cells/mL) of *Streptomyces* sp. MR14. Group 7 (CS) culture supernatant only: soil was treated with 10 mL of culture supernatant; Group 8 (CE) culture extract only: treated with culture extract of *Streptomyces* sp. MR14 (250 µg/mL). Along with each treatment, the tomato seedlings with true stage leaves were then transplanted singly into pots. Each treatment group was replicated three times and the pots were kept under natural conditions. Plants were watered daily and the wilting of tomato plant at pre-emergence growth stage was recorded after 45 days of treatments. The plants were uprooted and fresh and dry weights of tomato seedlings were recorded.

### Estimation of bacterial and fungal counts in the rhizosphere of the tomato plants

The rhizosphere soils of the treated plants were carefully sampled after 45 days. One gram of the soil was suspended in 9 mL of sterile distilled water and vortexed at high speed. The serial dilutions were prepared and aliquots of 0.1 mL from 10^−3^ dilution were spread on to Nutrient Agar (NA) amended with cycloheximide (50 µg/mL) and Potato Dextrose Agar (PDA) medium for bacterial and fungal counts, respectively. The NA and PDA plates were incubated at 37 °C and 28 °C, respectively. The CFUs (colony forming units) were counted after 24 h for bacteria and after 72 h for fungi.

### Statistical analysis

Data collected from the above experiments were subjected to statistical analysis where values were represented as their mean ± SD. To compare difference in means, one way analysis of variance (ANOVA) with Tukey’s post hoc test was performed using SPSS statistical analysis software (Version 20.0; IBM SPSS). Statistically significant difference was considered at *p* ≤ 0.05. Correlation analysis was also done using SPSS to determine the relationship between antifungal activity and biomass obtained.

## Results

### Characterization and identification of isolate MR14 using polyphasic approach

#### Morphological, biochemical and physiological characterization

**Fig. 1:**
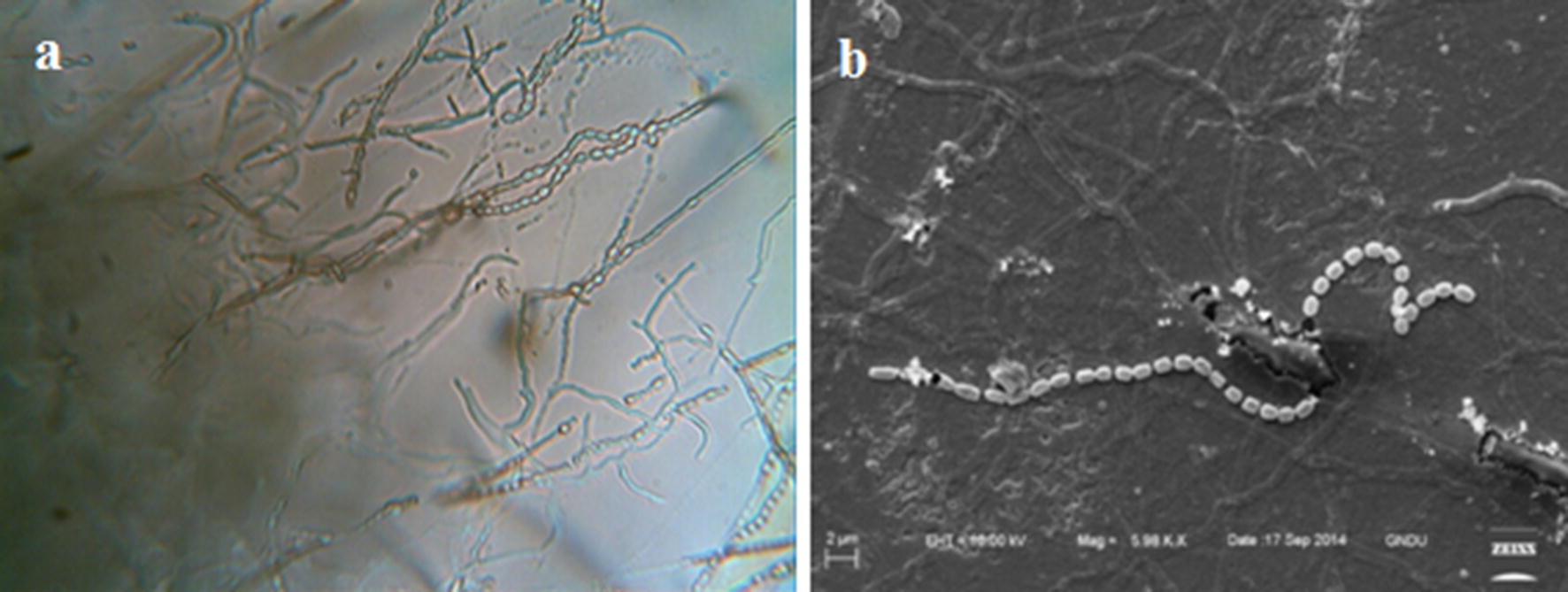
**a** Light micrograph at 100X showing rectus-flexibilis spore chains on aerial mycelium of *Streptomyces* sp. MR14 grown on Starch Casein Nitrate Agar for 5 days at 28 °C. **b** Electron microscopic view of aerial mycelium showing smooth surface of spores of *Streptomyces* sp. MR14 at 6000X

The actinobacterium MR14 displayed different colony morphological characteristics on various ISP media as shown in Table 1. Soluble pigment was not produced by the isolate on any of the media except ISP-7 medium which is the characteristic of melanin pigment production. The isolate showed cream sporulation and aerial mycelium and light yellow color substrate mycelium on the SCNA medium. Morphological characteristics such as spore chains of the isolate MR14 in the light microscope (100X) were observed as flexuous sporophores and placed in Rectus-Flexibilis (RF) group of *Streptomyces* (Fig. 1a). Micromorphological studies by SEM revealed chains of smooth surface spores on aerial mycelium, bearing 20–30 cylindrical spores (1.5–2.0 µm length and 1.5 µm width) (Fig. 1b).

**Table 1:** Cultural characteristics of *Streptomyces* sp. MR14 on ISP media and SCNA medium

Medium	Growth	Aerial mycelium	Substrate mycelium	Diffusible pigment
ISP-1	Very good	Cream	Brown	–
ISP-2	Very good	Creamish yellow	Light brown	–
ISP-3	–	–	–	–
ISP-4	Fair	Cream	Light brown	–
ISP-5	Very good	Cream	Grey	–
ISP-6	Good	Creamish yellow	Yellow	–
ISP-7	Very good	Light brown	Brown	Brown
SCNA	Good	Cream	Creamish yellow	–

The isolate grew well between 20 and 40 °C (optimum at 30 °C). It tolerated NaCl concentration up to 5.0%. It produced industrially important extracellular enzymes such as amylase, lipase, protease and urease by degrading their respective substrates starch, lipid, casein and urea. However, response to pectin, cellulose, citrate, chitin and gelatin degradation was negative. The strain neither reduced nitrate nor produced H_2_S. It gave positive results for both indole production and VP test. The strain was able to utilize most of the tested sugars as sole carbon source except xylose, arabinose and fructose. The maximum growth of MR14 was observed on medium containing starch and glycerol. Chemotaxonomic analysis of the isolate showed the presence of ll-diaminopimelic acid (ll-DAP) as the diagnostic amino acid in cell wall lysate and absence of characteristic sugars in whole cell hydrolysates (Table 2). Based on morphological, cultural and chemotaxonomical studies, strain MR14 belonged to genus *Streptomyces* which was further supported by 16S rRNA sequencing.

**Table 2:** Morphological, physiological and biochemical characteristics of *Streptomyces* sp. MR14

Characteristic	MR14
Spore mass	Cream
Spore chain	Recti-flexibilis
Spores shape	Cylindrical
Substrate mycelium	Creamish yellow
Aerial mycelium	Cream
Diffusible pigment	–
Sugar pattern	No characteristic sugar
Diaminopimelic acid	ll-DAP
Production of melanoid pigment on	
Peptone yeast extract agar (ISP-7) medium	+ (Brown)
Biochemical characteristics	
Amylase	+
Protease	+
Chitinase	–
Cellulase	–
Lipase	+
Gelatinase	–
Urease	+
Pectinase	–
H_2_S production	–
Nitrate reduction	–
Catalase	+
Oxidase	+
MR	–
VP	+
Citrate utilization	–
Indole production	+
Tolerance to NaCl	5%
Growth temperature	30 °C (20 to 35 °C)
Utilization of sugar	
Maltose	+
d-Glucose	+
Sucrose	+
Lactose	+
Inositol	+
d-Xylose	–
d-Fructose	–
Starch	++
Glycerol	++
Arabinose	–
Rhamnose	+
Raffinose	+

#### Phylogenetic and genomic analyses based on 16S rRNA Sequencing

**Fig. 2:**
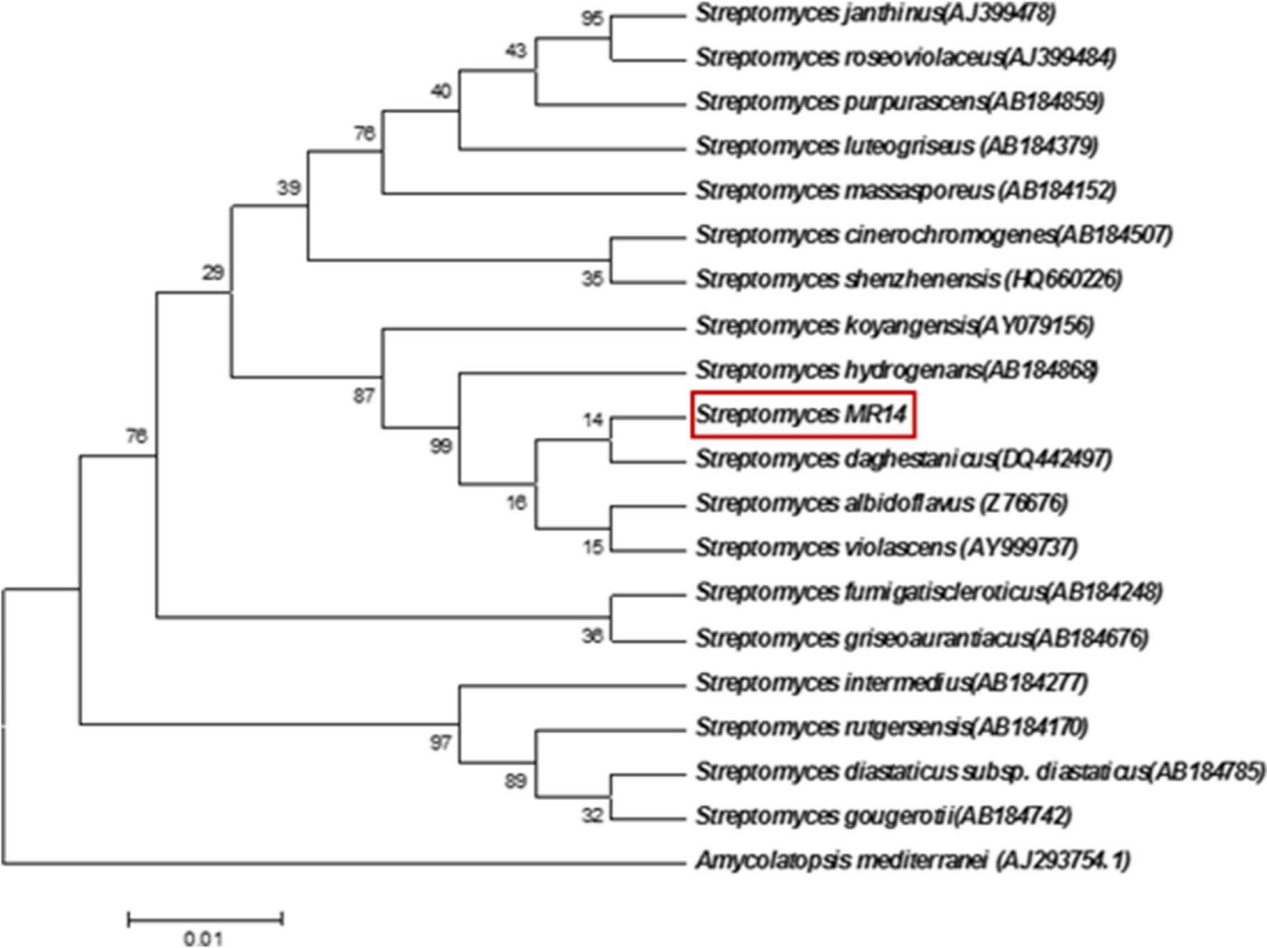
Neighbor-joining tree based on nearly complete 16S rRNA gene sequences showing the position of isolate *Streptomyces* sp. MR14 amongst its phylogenetic neighbors. Bootstrap values (expressed as percentages of 1000 replications) are shown at the nodes. *Amycolatopsis mediterranei* (AJ293754.1) was used as an outgroup. GenBank accession numbers are given in parentheses

**Fig. 3:**
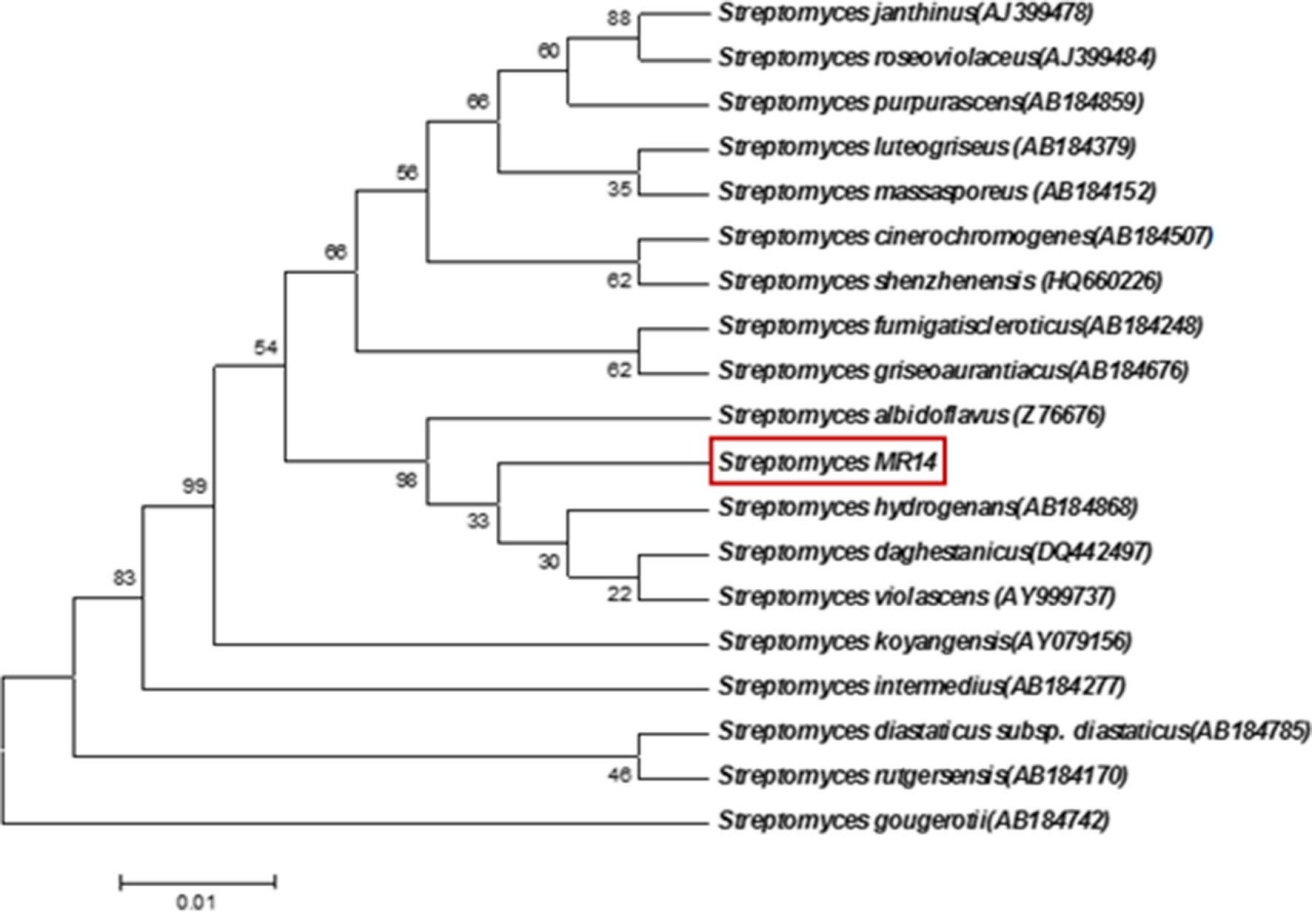
Maximum-parsimony phylogenetic tree based on 16S rRNA gene sequences of *Streptomyces* strains showing the position of isolate *Streptomyces* sp. MR14. Bootstrap values (expressed as percentages of 1000 replications) are shown at the nodes

16S rRNA gene sequence (1434 bp) of strain MR14 was compared with all available nucleotide sequences of other closely related *Streptomyces* species using the EzTaxon database. The data showed 100% sequence similarity with four *Streptomyces* spp. i.e. *Streptomyces violascens* ISP 5183 (T) AY999737, *Streptomyces hydrogenans* (AB184868), *Streptomyces daghestanicus* (DQ442497) and *Streptomyces albidoflavus* DSM 40455 (T) (Z76676). Further confirmation was done by constructing phylogenetic trees using neighbor-joining (Fig. 2) and maximum-parsimony algorithms (Fig. 3). In the phylogenetic tree constructed using the neighbor-joining method, MR14 formed clade with *S. daghestanicus* (DQ442497). However, the lower bootstrap value of 14 (< 50%) excludes the possibility of specific relatedness between the two species. This relationship was also supported by maximum parsimony tree where MR14 formed a phyletic branch different from all closely related species, showing low bootstrap value (34%). Thus on the basis of this, MR14 can be assigned as a new sp. of *Streptomyces,* designated as *Streptomyces* sp. MR14. The 16S rRNA gene sequence of the isolate MR14 has been deposited in the GenBank database under the accession number KY522669. The culture has been deposited in Microbial Type Culture Collection and Gene Bank (MTCC), Institute of Microbial Technology (IMTECH), Chandigarh (India), an International Depository Authority, and the accorded accession number is MTCC-12924.

### In vitro antifungal activity profile of *Streptomyces* sp. MR14 against fungal phytopathogens

**Fig. 4:**
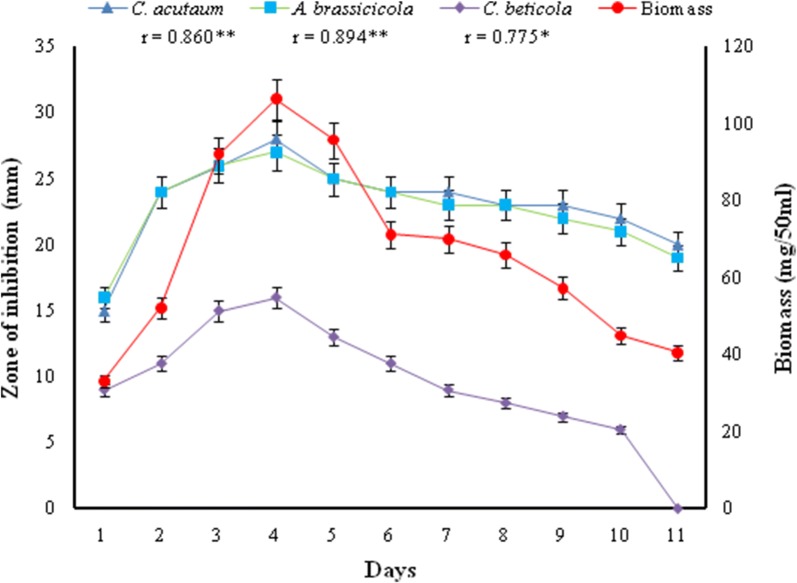
Growth and antifungal activity of *Streptomyces* sp. MR14 against different phytopathogens; **correlation is significant at 0.01% level; *correlation is significant at 0.05% level

In vitro activity profile demonstrated that the production of antifungal metabolites (in terms of inhibition zone) in culture broth by strain MR14 commenced on 1st day of incubation, reached the maximum after 4 days and then declined as the incubation was further extended. The maximum biomass production was also observed after 4 days of incubation which indicated positive correlation between the antifungal activity and growth (Fig. 4).

**Fig. 5:**
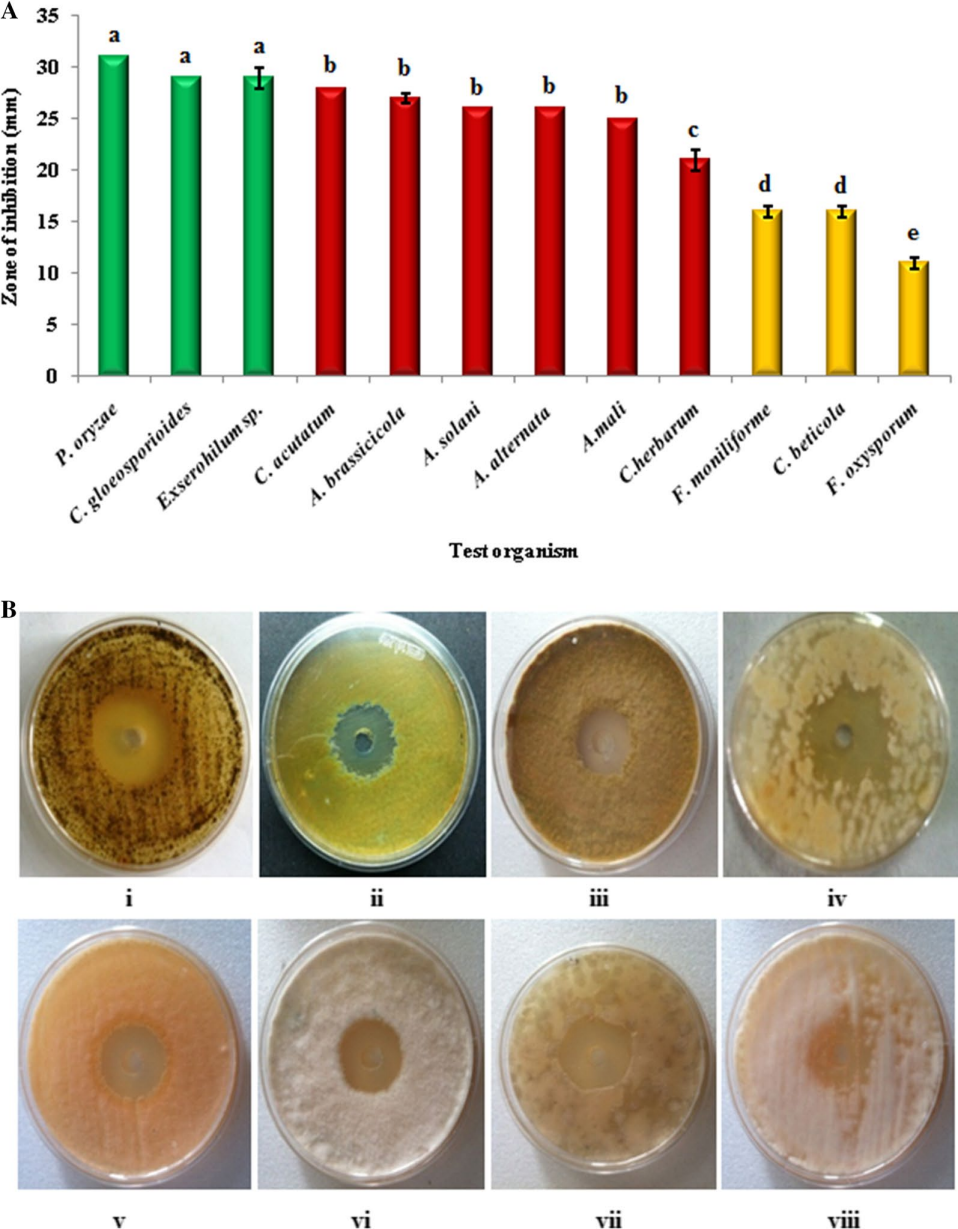
**A** Antifungal activity of *Streptomyces* sp. MR14 against different fungal phytopathogens; bars with different letters are statistically different (Tukey’s HSD, *p* ≤ 0.05). **B** The clear zones around the wells showing antifungal activity of *Streptomyces* sp. MR14 by well diffusion method against (i) *A. brassicicola* (ii) *A. mali* (iii) *A. alternata* (iv) *A. solani* (v) *C. acutatum* (vi) *C. gloeosporioides* (vii) *Exserohilum* sp. (viii) *F. moniliforme*

As the maximum antifungal activity was achieved at the 4th day of incubation, the spectrum of the antagonistic potential of the *Streptomyces* sp. MR14 was assessed using 4 days old culture supernatant against a variety of fungal phytopathogens. The strain displayed antifungal activity against all the tested fungal phytopathogens with inhibition zones ranging from 31 ± 0.0 to 11 ± 0.5 mm (Fig. 5). The highest activity, in terms of inhibition zone, was observed against *P. oryzae* (31 mm) closely followed by *Exserohilum* sp. and *C. gloeosporioides* (29 mm). Moderate activity was observed against *C. acutatum* (28 mm), *A. brassicicola* (27 mm), *A. alternata* (26 mm), *A. solani* (26 mm), *A. mali* (25 mm) and *C. herbarum* (21 mm), and weak activity was detected against *F. monilifome* (16 mm), *C. beticola* (16 mm) and *F. oxysporum* (11 mm).

### Stability of antifungal compounds in culture supernatant of *Streptomyces* sp. MR14

The stability of antifungal compounds present in the culture supernatant was checked for various physical stresses (Table 3). In terms of inhibition zone, the active metabolites responsible for the antifungal activity of *Streptomyces* sp. MR14 were found to be completely stable up to 50 °C for one hour, and loss of 22.86% in activity was observed at 70 °C. More than 50% activity retained after boiling for one hour and no activity was observed after autoclaving. The metabolites were also found to be photo stable because 97.14% of activity retained after exposure to UV light for 1 h. Antifungal compounds were also stable to enzyme proteinase K, showing 97.14% residual activity after treatment. More than 50% of residual antifungal activity was observed even at extreme acidic and basic pH of 2.0 and 14.0. Only 20% of loss in activity was observed even after 6 months storage of culture supernatant of *Streptomyces* sp. MR14 at 4 °C which indicated long shelf life of antifungal components at refrigeration temperature.

**Table 3:** Effect of temperature, pH, light and enzymatic treatments on the antifungal activity of culture supernatant of *Streptomyces* sp. MR14

Treatment	*Streptomyces* sp. MR14	
	Zone of inhibition (mm) against *F. moniliforme*	Residual activity (%)
Control (untreated)	35.0 ± 0.0	100.0
Heat treatment		
37 °C, 1 h	35.0 ± 0.1	100.0
50 °C, 1 h	34.0 ± 0.2	97.14
70 °C, 1 h	27.0 ± 1.0	77.14
100 °C, 1 h	18.0 ± 0.5	51.42
121 °C, 45 min	0.0 ± 0.0	0.0
Low temperature treatment		
− 20 °C, 1 h	35.0 ± 0.1	100.0
Enzymatic treatment		
Proteinase K	34.0 ± 0.0	97.14
Photostability		
Sunlight, 1 h	15.0 ± 0.5	42.85
UV light, I h	34.0 ± 0.1	97.14
pH tolerance		
pH 2.0	33.0 ± 0.5	94.28
pH 14.0	20.0 ± 1.0	57.14

Values represented as mean ± S.D (n = 3)

### Extraction of bioactive compound/s

For the recovery of bioactive compounds present in the culture supernatant of *Streptomyces* sp. MR14, solvents of different polarity were used. Among all the tested solvents, complete extraction of the bioactive metabolites was obtained using ethyl acetate at pH 5.0. Approximately 60 mg of solvent extract was obtained from 1 L of fermentation broth which was redissolved in 0.5% DMSO prior to the biocontrol assay.

### In vivo biocontrol of *F. moniliforme* causal organism of Fusarium wilt by *Streptomyces* sp. MR14 and its effect on plant growth promotion in tomato plants

**Fig. 6:**
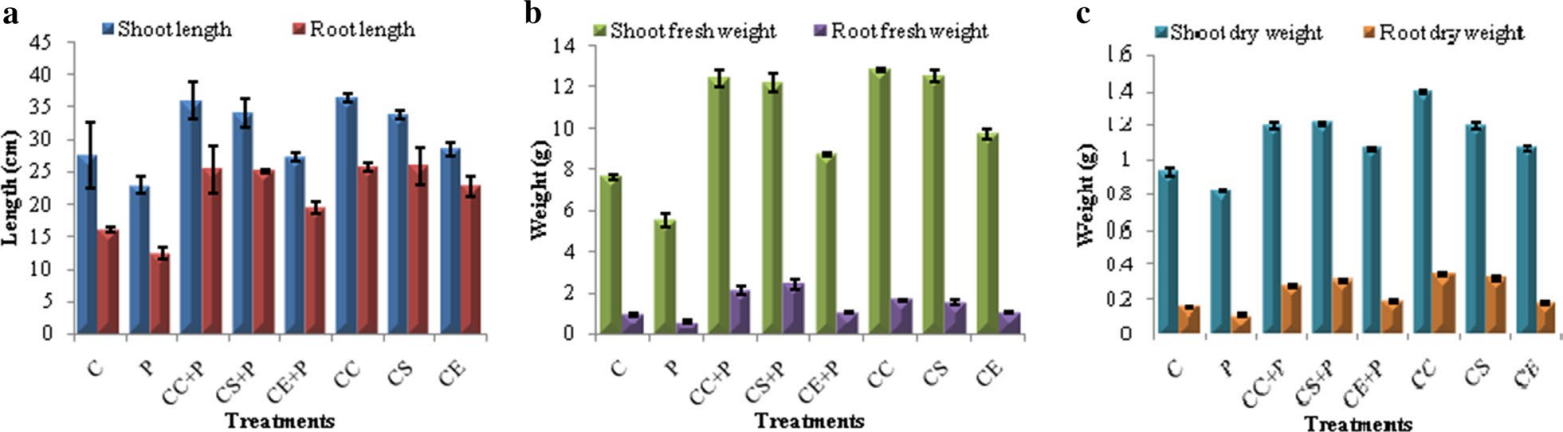
Effect of *Streptomyces* sp. MR14 and its metabolites as root treatments on *S. lycopersicum* (tomato) plants to control *F. moniliforme* causing Fusarium wilt disease **a** shoot and root lengths of plants, **b** fresh weights of shoots and roots, **c** dry weights of shoots and roots. Values were expressed in mean ± standard deviation; C, control (Water only); P, pathogen only; CC + P, *Streptomyces* sp. MR14 cells + pathogen; CS + P, MR14 supernatant + pathogen; CE + P, *Streptomyces* sp. MR14 solvent extract + pathogen; CC, MR14 cells only; CS, MR14 supernatant only; CE, solvent extract of strain MR14 only

**Fig. 7:**
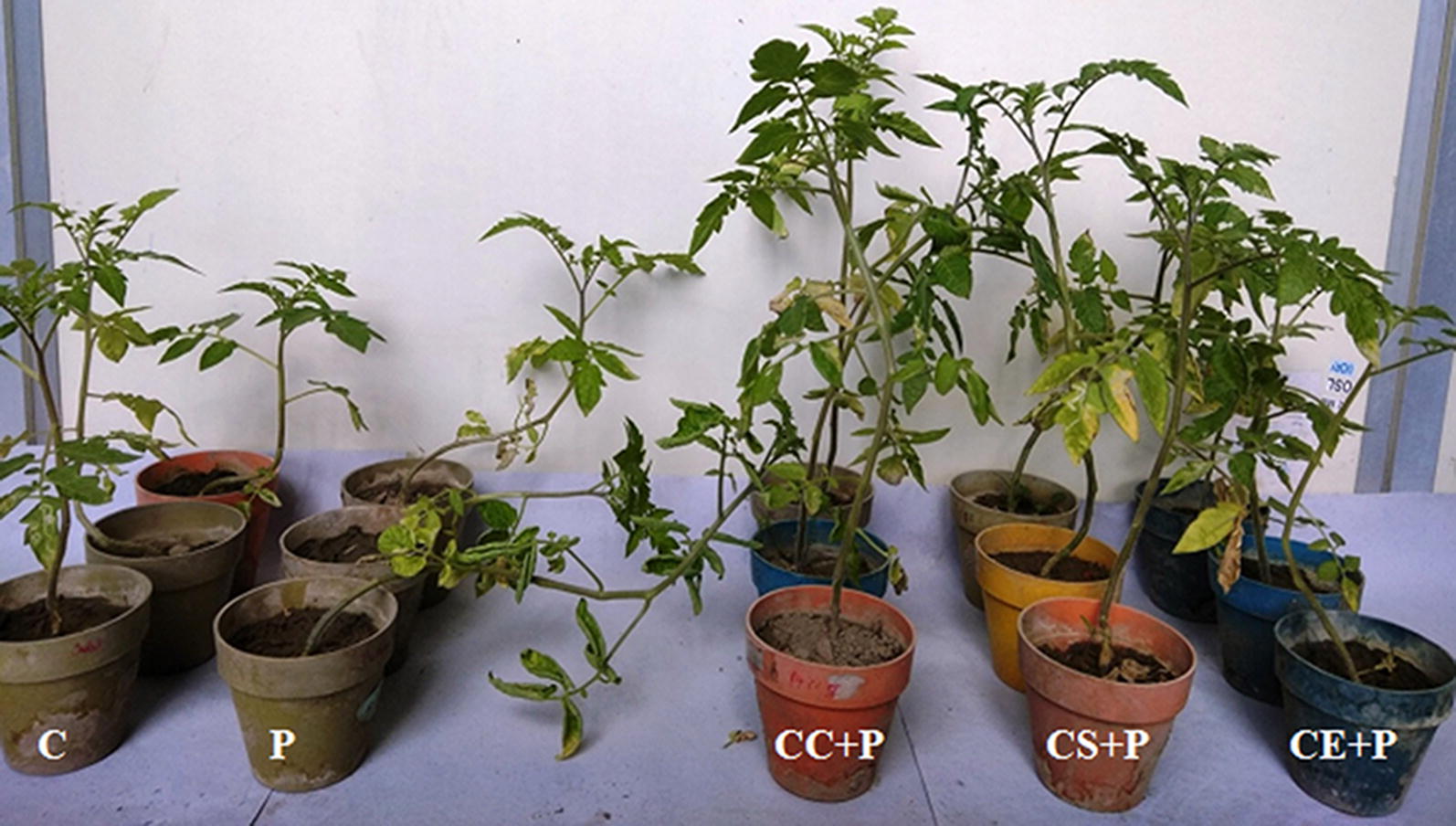
Effect of *Streptomyces* sp. MR14 and its metabolites on growth of *S. lycopersicum* (tomato) plants infested with *F. moniliforme*; C, control (water only); P, pathogen only; CC + P, *Streptomyces* sp. MR14 cells + pathogen; CS + P, MR14 supernatant + pathogen; CE + P, *Streptomyces* sp. MR14 solvent extract + pathogen

The pot experiments were carried out to evaluate in vivo biocontrol efficiency of strain MR14 against *F. moniliforme*. The data collected in terms of various agronomic traits of plant which are indicators of plant health and condition, such as shoot length, root length, shoot and root fresh and dry weights are shown in Table 4, Figs. 6, 7. The pots containing soil drenched with culture cells/culture supernatant/culture extract of strain MR14 and fungal pathogen significantly enhanced the root and shoot lengths, and fresh and dry weights of the tomato plants over the plants which were treated with pathogen only. Among the three groups, the soil treated with culture cells/supernatant and pathogen showed higher biocontrol efficiency than the culture extract and pathogen.

**Table 4:** Biocontrol and plant growth promoting effect of *Streptomyces* sp. MR14 on various growth traits of tomato seedlings infested with fungal phytopathogen *F. moniliforme*

Group	Shoot length (cm)	Root length (cm)	Shoot fresh weight (g)	Root fresh weight (g)	Shoot dry weight (g)	Root dry weight (g)
C	27.5 ± 5.1a	16.2 ± 0.4a	7.648 ± 0.1a	0.949 ± 0.07a	0.936 ± 0.02a	0.154 ± 0.007a
P	22.9 ± 1.4b	12.4 ± 0.9b	5.53 ± 0.3b	0.569 ± 0.08b	0.826 ± 0.01b	0.107 ± 0.005b
CC + P	35.9 ± 2.8ab (56.77)^1^	25.4 ± 3.7ab (104.84)^1^	12.421 ± 0.4ab (124.61)^1^	2.13 ± 0.2ab (274.34)^1^	1.194 ± 0.02ab (44.55)^1^	0.276 ± 0.009ab (157.94)^1^
CS + P	34.1 ± 2.2ab (48.91)^1^	25.2 ± 0.3ab (103.23)^1^	12.218 ± 0.4ab (120.94)^1^	2.399 ± 0.3ab (321.62)^1^	1.208 ± 0.01ab (46.25)^1^	0.299 ± 0.008ab (179.44)^1^
CE + P	27.4 ± 0.6 (19.65)^1^	19.5 ± 0.8b (57.26)^1^	8.744 ± 0.1ab (58.12)^1^	1.059 ± 0.02b (86.12)^1^	1.068 ± 0.009ab (29.3)^1^	0.185 ± 0.007ab (72.9)^1^
CC	36.4 ± 0.6ab (32.36)^2^	25.8 ± 0.7ab (59.26)^2^	12.822 ± 0.08ab (67.65)^2^	1.651 ± 0.02ab (73.97)^2^	1.404 ± 0.01ab (50.0)^2^	0.334 ± 0.007ab (116.88)^2^
CS	33.9 ± 0.6ab (23.27)^2^	25.9 ± 2.8ab (59.88)^2^	12.552 ± 0.3ab (64.12)^2^	1.557 ± 0.1ab (64.07)^2^	1.191 ± 0.02ab (27.24)^2^	0.315 ± 0.011ab (104.55)^2^
CE	28.5 ± 0.95 (3.64)^2^	22.8 ± 1.6ab (40.74)^2^	9.743 ± 0.2ab (27.39)^2^	1.053 ± 0.02b (10.96)^2^	1.072 ± 0.02ab (14.53)^2^	0.173 ± 0.011b (12.34)^2^

All data are presented as mean ± SD (n = 3). The same letters with in a column are significantly different (Tukey’s Test *p* ≤ 0.05). “a” indicates the statistical difference between the control and treated plants; “b” indicates the statistical difference between the pathogen infested and treated plants

*C* control, *P* pathogen, *CC* *+* *P* culture cells and pathogen, *CS* + *P* culture supernatant and pathogen, *CE* + *P* culture extract and pathogen, *CC* culture cells, *CS* culture supernatant, *CE* culture extract

^1^Values indicate percentage increase over pathogen infested plants

^2^Values indicate percentage increase over control plants

**Fig. 8:**
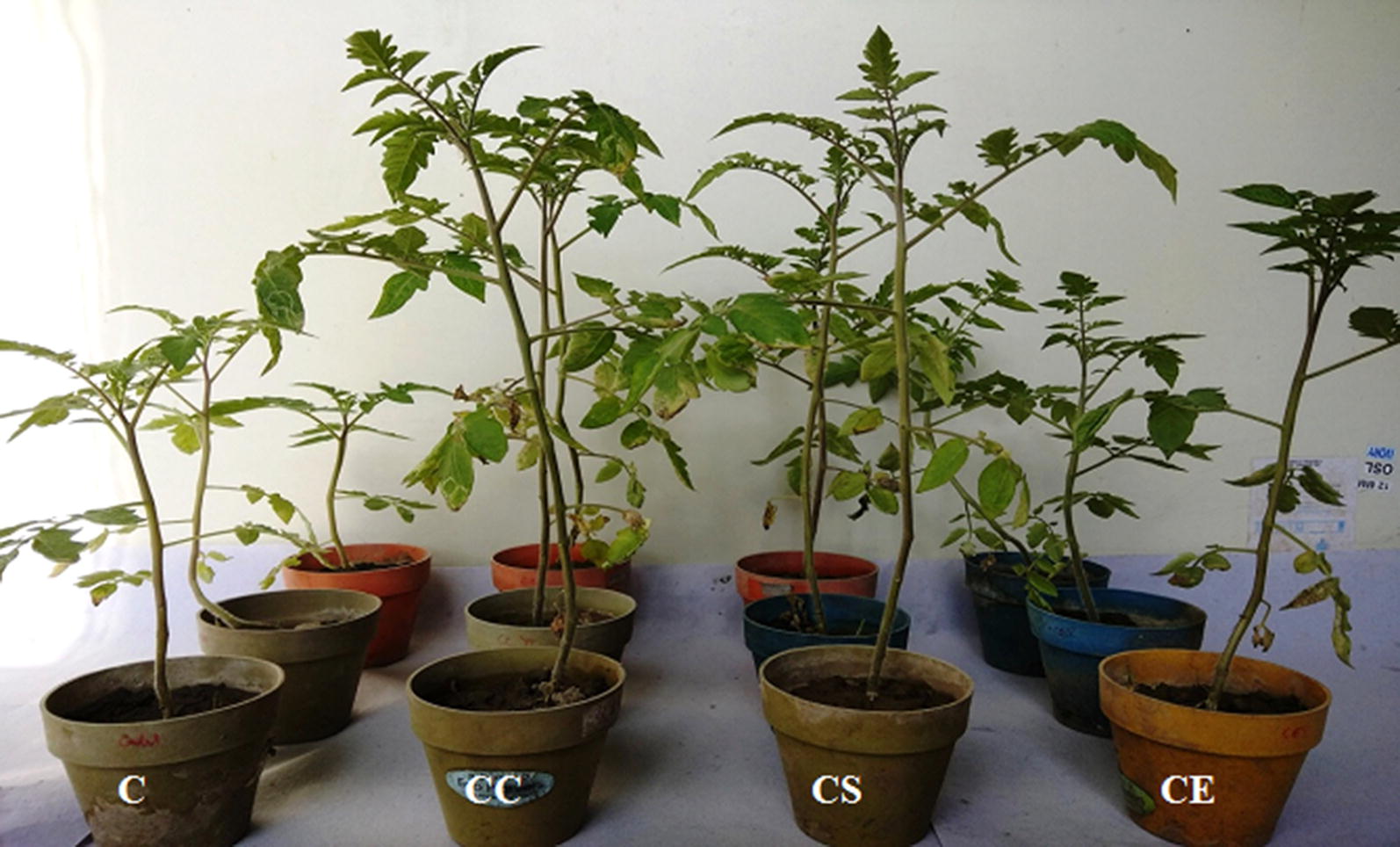
Plant growth promoting potential of *Streptomyces* sp. MR14 and its metabolites on growth of *S. lycopersicum* (tomato) plants; C (untreated plants, water only), CC (plants treated with culture cells only), CS (plants treated with culture supernatant only) and CE (plants treated with culture extract of strain MR14 only)

The strain MR14 not only controlled the disease caused by *F. moniliforme* but also promoted the growth of the tomato plants. The culture cells, culture supernatant and cell extract significantly increased the shoot (3.64 to 32.36%) and root (40.74 to 59.26%) lengths, fresh weights of shoots (27.39 to 67.65%) and roots (10.96 to 73.97%) and dry weights of shoots (14.53 to 50.0%) and roots (12.34 to 116.88%) over the control plants (Table 4, Figs. 6, 8). These data indicated biocontrol and plant growth promoting potential of *Streptomyces* sp. MR14 and its metabolites.

### Estimation of bacterial and fungal counts in the rhizosphere of the tomato plants

After 45 days, bacterial and fungal counts were determined in the rhizosphere of the treated plants (treated with culture cells/supernatant/extract along with pathogen). The results showed that the treatment with culture cells/supernatant/extract and pathogen significantly reduced the other competing bacterial and fungal counts along with the pathogen in the soil. The CFUs in control (water only) soil was very high as compared to the treated soils (Table 5).

**Table 5:** Bacterial and fungal counts in rhizosphere soils of control (water only) and different treated plants after 45 days

Treatment	Microbial count (10^4^ CFU/g)	
	Bacteria	Fungi
Control	146a	53a
CC + P	42ab	15ab
CS + P	34ac	12ac
CE + P	37ad	19ad

The same letters with in a column are significantly different (Tukey’s HSD *p* ≤ 0.05) and different letters with in a column are statistically insignificant different; Control, tomato plants treated with water only; CC + P, plants treated with *Streptomyces* sp. MR14 and pathogen; CS + P, plants treated with MR14 supernatant and pathogen; CE + P, plants treated with MR14 solvent extract and pathogen

## Discussion

In spite of the remarkable progress attained in plant breeding, and other disease management practices, loss in crop yield due to diseases caused by phytopathogens remains a major limiting factor in the agriculture growth globally. The losses are most likely to affect the tropical and developing countries. The food scarcity due to losses further creates the economical and health problems in community and hinders the development of the country. Much greater emphasis is required to control the phytopathogens by natural means. Biological control using *Streptomyces* spp. has the potential for the management of these diseases and to be further developed as BCAs and plant growth promoting agents (Hartman et al. 2009). Numerous streptomycete antagonists have been used to promote plant growth, and control soil-borne phytopathogens (Gopalakrishnan et al. 2011; Boukaew and Prasertsan 2014; Faheem et al. 2015).

In the present study, a potent streptomycete strain (MR14) isolated from mustard (*B. nigra*) rhizosphere was characterized through the polyphasic approach. Based on its cultural properties on different media, the strain belongs to the genus *Streptomyces*. Furthermore, the strain MR14 was also capable of producing a variety of enzymes such as amylase, lipase, protease and urease and could gain consideration in industrial sector. The bioactive compounds produced by the *Streptomyces* spp. are mainly secondary metabolites and greatly influenced by the availability of carbon sources given during the growth (Ser et al. 2016; Rani et al. 2018). The results indicated that the strain has the capacity to utilize a variety of substrates such as glucose, sucrose, lactose, inositol, starch, glycerol, rhamnose, raffinose and maltose. Chemotaxonomical markers (ll-DAP in cell wall and no characteristic sugar in whole cell hydrolysate) also supported that the strain MR14 belongs to the genus *Streptomyces*.

The low bootstrap value (14%) in comparative analysis done by constructing phylogenetic trees suggested the unrelatedness of strain MR14 with the closely related species *S. violascens* ISP 5183 (T) AY999737, *S. hydrogenans* (AB184868), *S. daghestanicus* (DQ442497) and *S. albidoflavus* DSM 40455 (T) (Z76676). Therefore, it might be assigned as a new sp. of *Streptomyces* and was supported by Sharma et al. (2014). They observed the low bootstrap value of novel *Streptomyces amritsarensis* sp. 2A with closely related type strains to which it showed very high i.e. 99.5–99.9% 16S rRNA sequence similarity. The novelty of the sp. was further proved by DNA–DNA hybridization. In contrast, Sahu et al. (2017) isolated a species of the novel genus *Allostreptomyces* showing the highest (99.0%) 16S rRNA sequence similarity with the type strain *Allostreptomyces psammosilenae* but with a very high bootstrap value (100%). However, DNA–DNA hybridization showed 54.5% relatedness with the type strain, proving the novelty of the species for which the name *Allostreptomyces indica* sp. nov. was proposed. Moreover, *Streptomyces* sp. MR14 is distinguished from the *S. daghestanicus* (which is phylogenetically more closest to MR14 and in the same clade) in terms of antimicrobial activity as the latter is not reported to possess any antimicrobial activity. *S. daghestanicus* is able to utilize xylose, fructose, citrate, gelatin and cellulose whereas MR14 does not. The novelty of the strain can be further confirmed by DNA- DNA hybridization.

In vitro antagonistic assay of culture supernatant done by well diffusion method revealed broad spectrum antifungal activity of the compound produced by *Streptomyces* sp. MR14, inhibiting wide range of fungal phytopathogens with varying degree of inhibition. The variation in antagonistic activity of strain could be related to the test pathogen. The culture supernatant showed pronounced effect against *P. oryzae* (31.0 ± 0.0), *Exserohilum* sp. and *C. gloeosporioides* (29 mm). The results demonstrated that *F. monilifome* (16 mm), *C. beticola* (16 mm) and *F. oxysporum* (11 mm) are more resistant as compared to other tested phytopathogens.

For the development of safe and effective formulation to be used as biocontrol and plant growth promoting agent in fields under varying climatic conditions in different regions, the bioactive compounds must be stable at various temperatures and in soils with different pH. The antifungal metabolites from *Streptomyces* sp. MR14 were found to be thermo and photo stable and active over extreme acidic and basic pH. This property is also quite useful during isolation, purification and processing of bioactive compounds from commercialization point of view.

In vitro disease suppressive potential of the strains does not ensure their use in the fields because various strains do not acclimatize or the bioactive compounds produced by a strain lose their activity in natural conditions (Li et al. 2011). Bhuiyan et al. (2003) observed that two bacterial isolates *Pseudomonas aeruginosa* and *Burkholderia cepacia* completely antagonized the *Claviceps africana*, the cause of ergot or sugary disease of sorghum (*Sorghum bicolor*) in vitro but failed to inhibit infection in vivo. Therefore, in vivo experiments were conducted to check the effect of *Streptomyces* sp. MR14 on disease suppression and promotion of the plant growth under natural conditions.

Tomato fruit, the product of *Solanum lycopersicum* L. plants, is one of the most consumed foods worldwide (Borrero et al. 2006). In 2014, India was the second largest country in production, accounting for about 10.95% worldwide (UN Food and Agriculture Organization Report 2016). Fusarium wilt in tomato mainly caused by *Fusarium oxysporum* f.sp. *lycopersici* is one of the major soil borne systemic diseases resulting severe reduction in yield (Larkin and Fravel 1998). There are numerous reports of the *Streptomyces* spp. with potential to control Fusarium wilt caused by *F. oxysporum* f.sp. *lycopersici*. In this particular study, the *Streptomyces* sp. MR14 was evaluated for the first time to control the Fusarium wilt caused by another species of genus i.e. *F. moniliforme*. *F. moniliforme* mainly affects the health of the plant by producing various toxins such as fusaric acid, fusarins, gibberellins, moniliformin, and fumonisins (Abbas et al. 1995). Inovejas and Divina (2018) reported the use of methanol extract and nanocomposite of *Trichoderma* sp. as a potential bio-control against *Fusarium moniliforme* in tomato plants.

In vivo pot experiments demonstrated that the soil infested with *F. moniliforme* and *Streptomyces* sp. MR14 cells/supernatant/solvent extract possessed the significant antagonistic ability to control the Fusarium wilt in tomato plants. The percentage increase in various growth traits over the pathogen infested plants clearly indicated the strong disease suppression by culture cells and culture supernatant and then followed by culture extract. The treatments altered the microbial community in the rhizosphere. Additionally, the strain MR14 produced significant amounts of IAA when grown in broth containing tryptophan, and siderophores and ammonia (Kaur et al. 2013). Further, the pot experiments determined the significant effect of *Streptomyces* sp. MR14 on various growth parameters of the plant such as root and shoot length, fresh and dry weights of the plants (3.64 to 116.88% increase as compared to the control). Therefore, the data obtained from in vivo experiments suggested that *Streptomyces* sp. MR14 might be used as a BCA and plant growth promoting agent in two formulations, one containing spores or mycelium and other containing antifungal metabolites produced by it.

The possible mechanism of various BCAs can be the production of antifungal compounds, hydrolytic cell wall degrading enzymes such as chitinase, β-1,3 glucanase and protease, siderophores, hyperparasitism and induced systemic resistance (De-Oliveira et al. 2010; Palaniyandi et al. 2013; Passari et al. 2015). They can further promote the growth of plants directly by enhancing the production of plant growth hormones such as indole-3-acetic acid (IAA) and solubilizing the inorganic phosphate and indirectly by the production of antifungal compounds (Hamdali et al. 2008; El-Tarabily et al. 2009; Goudjal et al. 2016). The results displayed that *Streptomyces* sp. MR14 cells or supernatant showed more pronounced effect on the biocontrol and plant growth promotion in tomato plant as compared to solvent extract. But, the reduction in microbial community was significantly same when soil was treated with culture cells/supernatant/extract and pathogen. The improvement in different plant growth parameters by culture supernatant or cells might be associated with the ability to produce IAA, ammonia and siderophores, and root colonization. These results suggest that *Streptomyces* sp. MR14 employ more than one possible mechanisms along with antibiosis to promote plant growth. In addition, *Streptomyces* sp. MR14 also exhibited antibacterial and nematicidal activities which further increase its effectiveness as biocontrol agent (unpublished observations). Therefore, it might be developed as safe multifunctional biopesticide against wide range of phytopathogens (fungi, bacteria and nematodes) and as bioinoculant to enhance plant growth.

This study concluded with highlighting the true biocontrol and plant growth promoting potential of a new potent rhizospheric actinobacterium *Streptomyces* sp. MR14. The strain produced stable antifungals as secondary metabolites in culture supernatant possessing broad spectrum activity against different phytopathogens. In vivo studies validated the biocontrol potential of the strain against Fusarium wilt caused by *F. moniliforme*, and also demonstrated the alteration in the microbial community structure. In addition, the strain significantly improved the growth and yield of tomato plants by reducing the risk of disease. This study opens the way for the development of new BCA and plant growth promoting agent using *Streptomyces* sp. for agricultural use and thus providing a platform for its usage at commercial level.

## Data Availability

All the data and materials have been provided in main manuscript.

## Abbreviations

BCAs: biocontrol agents
SCNA: Starch Casein Nitrate Agar
PDA: Potato Dextrose Agar
ll-DAP: ll-diaminopimelic acid
PCR: polymerase chain reaction
IMTECH: Institute of Microbial Technology
NA: Nutrient Agar
